# An 18-Year-old Prisoner with Abdominal Pain

**DOI:** 10.5811/cpcem.2018.3.37438

**Published:** 2018-06-25

**Authors:** T. Andrew Windsor, Anna Darby

**Affiliations:** * University of Maryland School of Medicine, Department of Emergency Medicine, Baltimore, Maryland; † Los Angeles County + USC Medical Center, Department of Emergency Medicine, Los Angeles, California

## CASE PRESENTATION (Anna Darby, MD, MPH)

An 18-year-old male presented to the emergency department (ED) with a complaint of severe abdominal pain for three days along with painful urination, vomiting, diarrhea and subjective fever and chills. The patient reported brief, severe, colicky episodes of mid and left upper quadrant (LUQ) abdominal pain that radiated to his testicles. He vomited several times because of the pain, which he stated began suddenly while he was lying down. Notably, the patient had recently got over a diarrheal illness a few days prior, followed by constipation, and had recurrence of one loose stool on the day of presentation. He denied any flank pain or back pain, and had never experienced anything like this current illness before.

The patient had no prior medical or surgical history, and had no known family history. His family lived in Honduras, but the patient was currently incarcerated. He was previously a one-pack-per-day smoker, drank alcohol one to two times per month, but denied drug use. Review of systems was negative for weight loss, headaches, chest pain, shortness of breath, melena, hematemesis, rashes, or joint swelling.

The vital signs were as follows: temperature 37.0°C orally, pulse 103 beats per minute, respiratory rate 11 breaths per minute (bpm), blood pressure 122/67 mmHg, and oxygen saturation 100% on room air. Physical examination revealed an alert young man intermittently doubled over in pain with spontaneous resolution. The heart was tachycardic and regular without murmurs, rubs or gallops. The lungs were clear bilaterally with normal work of breathing and no wheezes, rhonchi or rales. His abdomen was soft and non-distended with normoactive bowel sounds, but he demonstrated diffuse tenderness and guarding to palpation. He had no midline or costovertebral angle tenderness, and no ecchymoses were present on inspection of his back. His skin was warm, dry and without any obvious rashes. His neurological examination was grossly intact throughout. The patient was uncircumcised, and his right testicle was lying higher than his left, but neither was tender or swollen. No masses or inguinal hernias were appreciated in the groin.

**Image 1:**
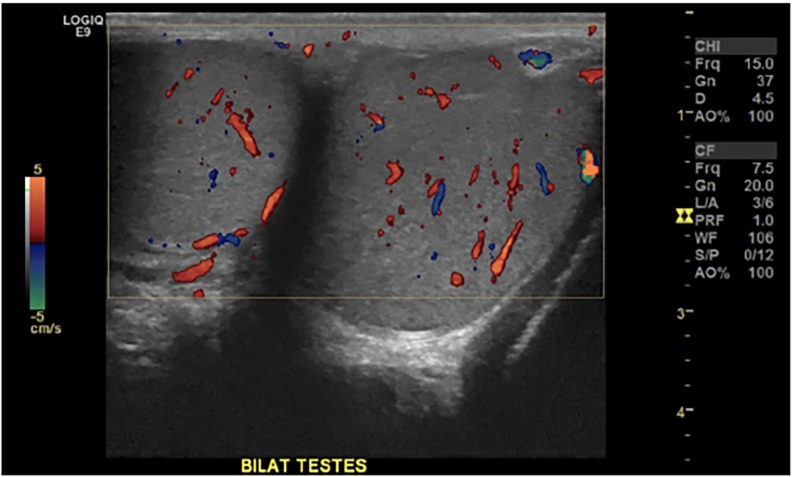
The patient’s normal scrotal ultrasound.

Laboratory studies were ordered (Tables 1–3), and a point-of-care focused assessment with sonography for trauma (FAST) exam and gallbladder ultrasound were normal. The patient had a formal scrotal ultrasound performed. (Image 1).

The patient’s pain was initially well controlled with hydrocodone/acetaminophen and non-steroidal anti-inflammatories; however, as more laboratory and imaging studies resulted, the patient continued to have intermittent pain episodes requiring morphine for analgesia. After two to three hours, the pain crises appeared more severe and the patient became more tachypneic to 18 bpm. It was at that point that an additional study was ordered, and the diagnosis was subsequently made.

## CASE DISCUSSION (T. Andrew Windsor, MD)

Often, as we work through a differential we start broadly and narrow our way through our previously great ideas that have seemingly gone south as we blend the story together with our objective data. I’ll walk you through my process. To start, let’s go back and summarize the case: an 18-year-old male with three days of intermittent abdominal pain that radiated to the testicles. That gave me some initial confidence based on pattern recognition, until I kept reading and saw that the patient had no flank or back pain. That would make something simple and common like a kidney stone unlikely.

He did have a recent diarrheal illness, and on the day of presentation he had vomiting and diarrhea, although he had also apparently had constipation for about three days prior to this. He was an occasional drinker, but I doubt he had been drinking much in jail unless it was something like “toilet wine.” While I know he was in jail and originally from Honduras, I don’t know how long he had been incarcerated or when he was last in his home country. Presuming this presentation was in the United States, I will assume he’d been in jail for less than a year since he was only 18. While it is not impossible that he had been previously or recently exposed to a disease such as tuberculosis, my feeling is that disseminated disease would be less likely based on his previously well state.

At the University of Maryland, one mnemonic that many of us teach to our students and interns for getting complete histories regarding pain is OLDCAAAR (Onset Location Duration Character Associated symptoms Aggravating factors Alleviating factors Radiation), and I think it’s good to go back to this to get an idea of his description of the abdominal pain. Its Onset occurred about three days ago, suddenly, at rest, after his diarrheal illness stopped and his constipation started. The Location was described in the LUQ and mid-abdomen. I don’t know if that means mid-upper abdomen as in the epigastrium, or mid-abdomen as in the periumbilical region. As you’ll see, those can mean very different things. The Duration was brief and the Character was severe and colicky but self-resolving. It was Associated with vomiting, diarrhea and dysuria (which is somewhat curious), fever and chills, as well as tachypnea. I don’t really know if anything in particular Aggravated the pain, as this information wasn’t included in the history, and it seemed to get better on its own without any particular Alleviating factors. The pain Radiated to the testicles.

His laboratory studies showed a normal chemistry, a complete blood count only remarkable for a leukocytosis, and a urine with some microscopic hematuria and a fair amount of squamous epithelial cells. I was informed that the patient was uncircumcised, which could possibly be the cause of the higher-than-expected number of squamous cells. There was trace urinary protein, but for now I think this is of uncertain significance.

On exam, we see that the patient is afebrile but mildly tachycardic, which is not surprising given the situation and his pain. He has diffuse abdominal tenderness with some guarding, but he does not seem particularly peritoneal. No back pain or costovertebral angle tenderness was noted, so it is less likely to be a renal or urologic problem. About 60% of men will have the left testicle lie lower than the right, so in the absence of tenderness or swelling that is a normal exam finding. Cremasteric reflex should, however, be verified.

The imaging studies presented, including his ultrasounds, were normal. I’m not 100% sure why he had a right upper quadrant ultrasound when his complaint was LUQ and mid-abdominal pain, but perhaps it was done for the sake of completeness. I saw no gallstones, gallbladder wall thickening, or pericholecystic fluid on the images I was provided. Both testicles have normal flow, so it is unlikely to be torsion. It is important to note that intermittent testicular torsion can have a normal flow when the testicles are not actively torsed, but given that his symptoms worsened prior to presentation, this diagnosis is unlikely.

I’m still left with a few things I want to know. For instance, what is up with the dysuria? Does eating make the pain worse? Are his pulses normal, does he have any organomegaly, are there any other laboratory studies that were sent, and when did he go to jail? As it is in real-life emergency, we are not always provided with every bit of information we would like, but working with what we have we can start with a differential.

**Image 2:**
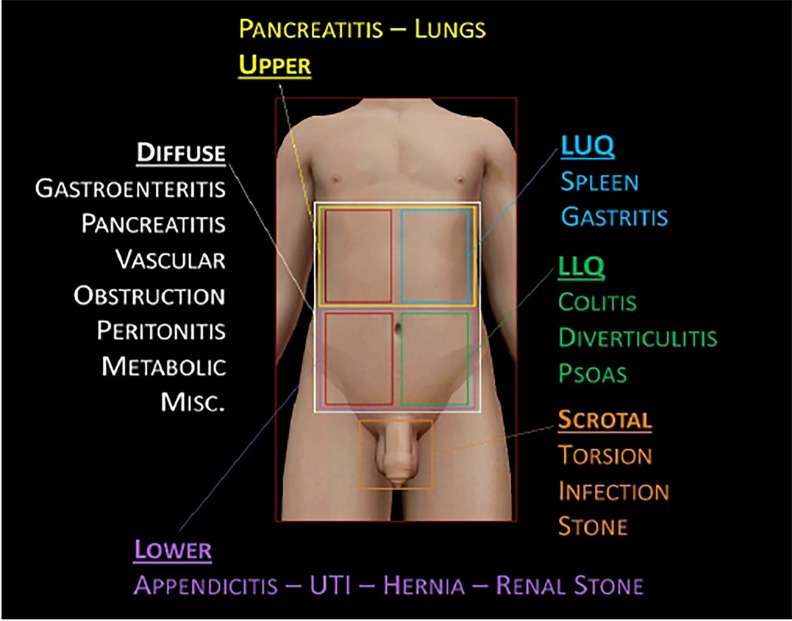
Example differential diagnosis for abdominal pain based on location of the pain.^1^ *LUQ*, left upper quadrant; *LLQ*, left lower quadrant; *MISC*, miscellaneous; *UTI*, urinary tract infection.

In general, with abdominal pain we usually think of quadrants (Image 2), and in this case we’re also dealing with scrotal pain, which I’ll consider a “fifth” quadrant, if you will. Based on location of the pain, we can form a basic differential diagnosis with which to start. Now, just from the nature of this being a clinicopathological case (CPC), there are a few things we can knock off the list, including simple stomach issues such as gastroenteritis. However, when considering a diagnosis, especially with abdominal pain, it’s important to be sensitive to the fact that there are certain things that tend to be more common in different age groups. For example, it’s very unlikely a child would have diverticulitis; on the other hand, you would very probably pause to consider aortic pathology before diagnosing grandpa with a first-time kidney stone.

Our patient falls between the child and adult age groups, so we’ll have to keep that in mind. There are a few things that we can eliminate rapidly. His blood work points away from anything metabolic, and his urine is negative for infection. Although he has mid-abdominal pain, nothing points towards early appendicitis. There are no hernias on exam, and as before no flank pain for a renal stone, even though there is radiation to the scrotum. We already discussed that the testicular exam was normal with normal flow on ultrasound. It would be unlikely for the symptoms to be due to a psoas abscess, and he would be very young for diverticulitis. Although the patient has become more tachypneic, he has normal lung sounds. I think it is unlikely to be a primary lung source; instead, it could have been from pain or something pushing up on his diaphragm.

Since we’ve narrowed down a bit, let’s focus in and look at individual features again. To recap, the patient has LUQ and mid-abdominal pain that radiates to the testicles and he has diffuse tenderness on exam. The way we feel pain in the abdomen is by several different mechanisms, including somatic pain, visceral pain and referred pain. The different areas involved probably reflect a bit of all three. Somatic pain is generally experienced from irritation of the parietal peritoneum. That pain is well localized – as in his LUQ pain, for example.

Visceral pain, on the other hand, is vague, deep and poorly localized, and is often felt in conjunction with or referred from an area of embryonic development. This is why you can have diffuse abdominal pain before localizing to the right lower quadrant in something like appendicitis. Referred pain is felt in a remote place from the source, for instance, renal colic causing pain in the groin; it often happens because nerves providing sensory information from different areas converge at their entry to the spinal cord. Our patient has pain that radiates to the testicles; so when we think about how the scrotum is innervated, we know there are both somatic and sympathetic nerves that do that job.

As opposed to the somatic nerves, which originate from the upper lumbar spine, the sympathetic nerves can be more visceral, cover a larger area and interact with other plexuses. Several organs have been associated with testicular pain, including the stomach, pancreas, kidneys/ureters and intestines. Considering the stomach, the patient does have nausea and vomiting, and it is in the right anatomic area for the pain; but unless the patient had perforated an ulcer, I don’t see why he would have diffuse tenderness, and he is neither febrile nor seemed peritoneal on exam. So, I’m taking that off my list. The pancreas is also in the right anatomical area, the symptomatology was reasonable, and he does drink. However, most case reports of pancreatic pain referred to the testicles is associated with scrotal edema,^2^ the thought being that pancreatic fluid drains down through the retroperitoneal space, and his testicles were not tender. So that’s off my list.

Earlier we established that there is unlikely to be direct involvement of the urinary system and there are no kidney issues, so those are gone as primary sources. All that’s left is the intestines, and a lot of the patient’s symptoms make sense for this. The way his pain is described makes it hard to localize, and he had the recent diarrheal illness followed by constipation, then nausea and vomiting. To me, this progression points to a possible obstructive process. While diffuse tenderness isn’t diagnostic, it does fit. What I had a hard time wrapping my head around was the dysuria. Was this from ureteral or bladder irritation, or something else?

Going back to our basic list, I think I would be remiss to ignore the spleen for someone who presents with LUQ pain, even though the patient’s symptoms don’t really fit. There was no report of any trauma, and the colicky pain would be unusual for splenic pathology. I wasn’t made aware of any organomegaly, but that would be important to check for. That being said, there is a rare condition called “wandering spleen” due to laxity in the suspensory ligaments. Other than possibly causing migrating pain, the pattern still doesn’t fit this diagnosis. Furthermore, that condition does not present acutely.

What about vascular issues? The patient experiences intense waves of pain; so could this be some sort of ischemic process? Maybe there’s an underlying structural abnormality or vasculitis? What points away from ischemic pain, within the limited data we have, is that the patient is young, he had a normal lactate, at least initially, and he had no pain out of proportion to exam. It would have to have been transient ischemia if at all. Based on the patient’s pain, the most likely culprits would be upper vascular branches such as the celiac, superior mesenteric artery (SMA) or renal artery, and if involved at all, the symptoms would most likely be from spasm or compression. Two examples include median arcuate ligament syndrome (MALS),^3^ which is caused by compression of the celiac trunk by the median arcuate ligament, and nutcracker syndrome, which is caused by compression of the renal vein from the SMA.^4^ MALS tends to be more constant, have less of a sudden onset, worsen with food, etc. Nutcracker syndrome is more commonly associated with gross hematuria, and because the left gonadal vein comes off of the renal vein, I would expect the testicular exam to have shown a varicocele or altered flow on the ultrasound.

Given the patient’s age, it would be extremely unlikely to be an abdominal aortic aneurysm or dissection. If we consider inherent vascular issues such as vasculitis, we’d be looking for one that could cause a constellation of symptoms consistent with the patient’s presentation. There are a number of possible small and large vessel vasculitides such asTakayasu’s or lupus mesenteric vasculitis, but no syndrome fits nicely except for one in particular, Henoch-Schönlein purpura (HSP).

HSP, also known as IgA vasculitis, fits with some things from our patient’s presentation, such as his colicky abdominal pain as well as a recent gastrointestinal (GI) illness, although we don’t know the specific source. The patient had the expected microscopic hematuria and proteinuria, and congruent with HSP (and unlike other purpuric diseases), he had normal platelets. Unfortunately, the patient doesn’t have other usual symptoms like arthritis or a rash. However, not everyone presents like this, and HSP may first have atypical symptoms before the typical ones such as rash manifest.^5^

Overall I’m not sure that we can completely exclude a vascular problem yet, so we’ll move on to some miscellaneous causes because I’m beginning to feel confident that this was more directly related to the bowels than anything else. Because the patient is a prisoner, you’d have to at least consider an ingested or inserted foreign body, but nothing in his history suggests that. Could he have had a toxic bite or exposure to something? Possibly, but without geographical information available to me while looking at the case, it would be hard to pinpoint this. He did drink, and he was 18, so it would be remiss not to consider a toxic alcohol ingestion; however, his metabolic panel was normal and he doesn’t really fit a toxidrome. He has no signs of trauma on exam, and I don’t get any indication that he’s malingering.

Since this is a CPC, we’ll briefly address a couple of zebras. There have been case reports of pulmonary embolism presenting with abdominal pain,^6^,^7^ but he is not hypoxic, he has no leg swelling, and he does not have any risk factors. Furthermore, the pain shouldn’t be colicky. I’d also expect that if he had upper quadrant pain from possible lung infarct-related irritation of the diaphragm, it would radiate to the shoulder rather than the testicles. The possibility of acute intermittent porphyria (AIP) was something that I entertained for a while because it fits the picture of having abdominal pain with dysuria; but he did not have any of the neurologic findings or psychiatric symptoms, and the attacks from AIP tend to result in more constant pain.^8^ Furthermore, the specific finding of AIP is rarely an ED diagnosis unless you could do an on-the-spot porphobilinogen test, which isn’t available in our department. I suppose, though, you could take the urine sample to a windowsill and look for darkening after ultraviolet light exposure.

I think we have narrowed down the pain source to vascular or bowel pathology. With the diarrhea a presenting feature, an infectious cause is possible. However, the patient does not have any fevers, and has not had any bloody or mucous stool. His diarrhea had improved and there was no reported recent travel; so he does not fit any of the patterns of the well-known infectious diarrheas. What does fit, because of the severe waves of pain, is some sort of obstructive process. I like the way intussusception, although rare in an adult, more closely fits the patient’s presentation, more so than something like a volvulus or a functional problem. The patient experienced brief, severe episodes that self-resolved, which could be due to telescoping of bowel in and out. Although the most common location is usually ileocecal, and one might expect right lower quadrant pain, the pain from intussusception can occur anywhere, and as previously discussed the pain sensations we experience can be vague or poorly localized if visceral. Furthermore, an obstruction anywhere can cause pain upstream due to dilation. We typically think of “currant jelly stool” as part of the classic triad of intussusception, along with vomiting and abdominal pain. But keep in mind, “classic” in medicine often actually means “you probably won’t see it.” That triad is present in only around a third of patients, and bleeding is a late finding, less common in adults.

Does it fit? I think it does, especially the severe, brief nature, and the fact that it followed a diarrheal illness. What I’m still having a hard time fitting into the picture is the dysuria and hematuria. I suspect this would either have been reactive or from a more widespread condition like vasculitis. Intussusception usually has a lead point, so absent cancer or previous scar tissue, I went back to my list of vasculitides as a possible explanation, and HSP kept coming up with symptoms similar to our patient’s. It is well known to cause intussusception in kids, and I found a number of reports^9^–^10^ of HSP presenting as intussusception in adults.

That being said, my test of choice is computed tomography (CT) of the abdomen and pelvis as there is no way to avoid scanning this patient based on his worsening presentation. I’m confident he has an obstructive bowel process. Any other diagnosis would be one of exclusion after making sure there isn’t a mechanical cause. Some might suggest starting with an ultrasound and I like that idea; however, it would depend on his habitus, and if the ultrasound was inconclusive a CT would follow anyway. I only have enough information to diagnose intussusception, not HSP, but he should have close surveillance for HSP with a thorough skin exam, and a high index of suspicion. My final diagnosis is intussusception, with CT as the test of choice.

## CASE OUTCOME (Anna Darby, MD, MPH)

In order to provide appropriate care for this patient, it became clear that more imaging would be required. At the time of presentation, his complaints seemed to be concerning for a genitourinary (GU) source: nephrolithiasis, testicular torsion or GU infection all seemed plausible. However, with a negative testicular ultrasound and worsening pain, a CT of the abdomen and pelvis was ordered and revealed the diagnosis of ileocolic intussusception. Once the CT resulted, an emergent surgery consult was placed. The patient was rushed to the operating room and underwent right hemicolectomy with part of the terminal ileum, ascending colon, and transverse colon removed. The pathology report showed ulceration with extensive acute inflammation, and reactive lymphoid hyperplasia with acute lymphadenitis. Luckily, this patient did well after the operation and was discharged on postoperative day three without any complications.

## RESIDENT DISCUSSION

Intussusception is classically described as a proximal segment of GI tract “telescoping” into a more distal portion of the bowel. This process is traditionally classified based on location and can lead to bowel obstruction proximally, gut ischemia, or perforation. Although intussusception is predominantly a pediatric diagnosis (the classic triad taught in most medical schools being intermittent abdominal pain, “red currant jelly” stool, and a palpable mass), adults account for about 5% of all cases of intussusception. The classic triad is much less likely to occur in adults than in children.^11^

The majority of all adult cases are due to a “lead point” lesion, meaning some kind of physical abnormality that makes a portion of the bowel more likely to experience the telescoping. Among adults, tumors (~50%), adhesions, and lesions from inflammatory bowel disease are the most common lead points thought to trigger intussusception. These are most commonly enteroenteric in location, with the most likely presenting symptom being abdominal pain, followed by changes in bowel patterns, vomiting, rectal bleeding and/or melena.^12^

In this case, we are reminded why adult intussusception can be such a difficult diagnosis to make. Although our patient did exhibit abdominal pain as well as changes in bowel patterns and vomiting, he did not have the “classic” signs of pediatric intussusception and thus initially was somewhat of a medical mystery. Besides age, his leukocytosis, testicular pain, and hematuria led us down several erroneous diagnostic paths before we were able to make the correct diagnosis. In hindsight, our patient’s pathology results of lymphoid hyperplasia were consistent with his recent GI infection, and in fact likely served as the lead point in this particular case.

**Image 3:**
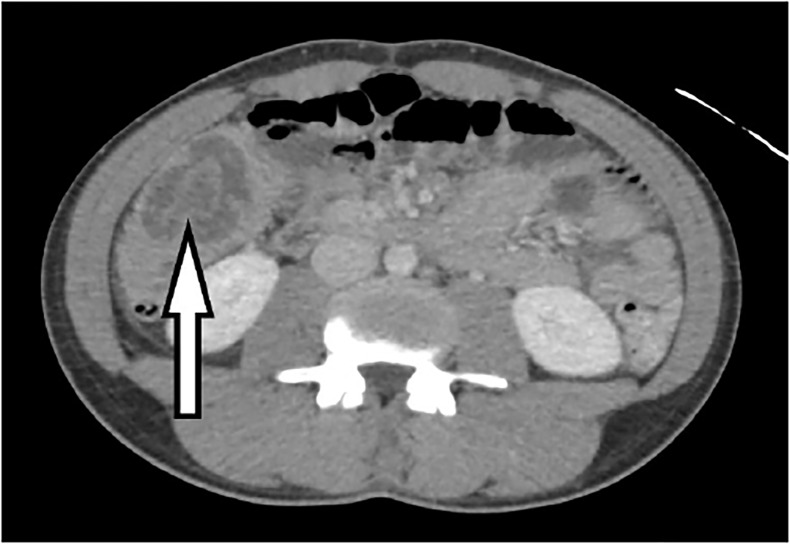
Example of the “target” sign (arrow) of intussusception as seen on the patient’s computed tomography.

Misdiagnosis is somewhat common in adult intussusception. Up to 20–30% of patients may be asymptomatic, and the aforementioned symptoms are somewhat nonspecific.^13^ Because delays in diagnosis of adult intussusception have proven to increase morbidity a high clinical suspicion is often key. As we saw in our case, CT is the gold standard for diagnosis and will typically reveal a “target”-like mass, which is due to the overlap of the inner and outer portions of the telescoped bowel (Image 3). Plain radiographs have neither the sensitivity nor specificity to reliably pinpoint the lesion but may show signs of obstruction and oral or enema contrast may identify the location of the intussusception. Ultrasound is used commonly for diagnosis in children, but may be limited due to bowel gas from an obstruction or body habitus in adults.

Unlike children, who are generally treated with either a barium or air enema, management of adult intussusception has traditionally been surgical because of the high proportion of pathologic lead points. Resection of the lead point and any ischemic area is the traditional surgical goal. There is, however, a more recent acknowledgment that with the development and increased use of better diagnostic imaging, more idiopathic or incidental intussusceptions are being diagnosed and that some adult patients may be treated non-surgically.^14^ If adult intussusception patients display obstructive symptoms, GI bleeding, or have a palpable mass, surgical exploration may still be the most appropriate option.^15^

## FINAL DIAGNOSIS

Intussusception

## KEY TEACHING POINTS

- Adult patients with intussusception can present with a wide variety of symptoms and do not abide by the classically taught, pediatric intussusception triad. Up to one third of adults with the disease may be asymptomatic.
- Delays in diagnosis of adult intussusception have proven to increase morbidity, so a high clinical suspicion is key with concerning symptoms.
- CT is the gold standard for diagnosis of intussusception in adults.
- While traditional treatment of adult intussusception was once strictly surgical, for patients with more mild disease there may now be a role for non-operative management. Any patient with obstructive symptoms, bleeding, or signs of a mass should still undergo surgical exploration.

Documented patient informed consent and/or Institutional Review Board approval has been obtained and filed for publication of this case report.

## Figures and Tables

**Table 1:** Complete blood count results for patient presenting with severe abdominal pain.

White blood cells	18.1K/mL	Reference (Ref.) 4–10K/mL
Hemoglobin	14.8 g/dL	Ref. 13–17 g/dL
Hematocrit	43.10%	Ref. 40–52%
Platelets	232K/mL	Ref. 150–400K/mL

**Table 2:** Serum chemistry results

Sodium	139 mEq/L	Ref. 135–145 mEq/L
Potassium	4.3 mEq/L	Ref. 3.5–5 mEq/L
Chloride	99 mEq/L	Ref. 95–105 mEq/L
Bicarbonate	27 mEq/L	Ref. 23–29 mEq/L
Blood urea nitrogen	10 mg/dL	Ref. 8–21 mg/dL
Creatinine	0.64 mg/dL	Ref. 0.8–1.3 mg/dL
Glucose	119 mg/dL	Ref. 65–110 mg/dL
Total protein	7.6 g/dL	Ref. 6–8 g/dL
Albumin	4.6 g/dL	Ref. 3.5–5 g/dL
Alkaline phosphatase	68 IU/L	Ref. 50–100 IU/L
Alanine aminotransferase	27 IU/L	Ref. 5–30 IU/L
Aspartate aminotransferase	33 IU/L	Ref. 5–30 IU/L
Total bilirubin	0.4 mg/dL	Ref. 0.1–1.2 mg/dL
Direct bilirubin	0.1 mg/dL	Ref. 0.1–0.4 mg/dL
Magnesium	2.0 mEq/L	Ref. 1.5–2 mEq/L
Lactate	2.0 mmol/L	Ref. 0.5–1 mmol/L

*Ref*, reference.

**Table 3:** Urinalysis results

Color/clarity	Straw, clear	Ref. yellow, clear
Specific gravity	1.028	Ref. 1.005–1.025
pH 6.5	6.5	Ref. 4.5–8
Protein	Trace	Ref. Negative
Glucose	Negative	Ref. Negative
Ketones	Negative	Ref. Negative
Bilirubin	Negative	Ref. Negative
Urobilinogen	0.2 EU/dL	Ref. 0.1–1 EU/dL
Leukocytes	Negative	Ref. Negative
Nitrite	Negative	Ref. Negative
White blood cells	0–3/High powered field (HPF)	Ref. 0–3/HPF
Red blood cells	11–25/HPF	Ref. 0–3/HPF
Bacteria	None	Ref. None
Squamous epithelial cells	16–30/Low powered field (LPF)	Ref. 0–5/LPF
Hyaline casts	0–4/LPF	Ref. 0–4/LPF

*Ref*, reference.
